# Supplementary material for: Prolonged experimental CD4+ T-cell depletion does not cause disease progression in SIV-infected African green monkeys
Source: Nat Commun. 2023 Feb 22;14:979. doi: 10.1038/s41467-023-36379-2 (PMC9946951; doi:10.1038/s41467-023-36379-2)
Supplement: Supplementary file 1 — Supplementary Information [file 41467_2023_36379_MOESM1_ESM.pdf]

Supplementary Fig. S1. Dynamics of different biomarkers of toxicity after administration of the CD4-depleting monoclonal antibody CD4R1

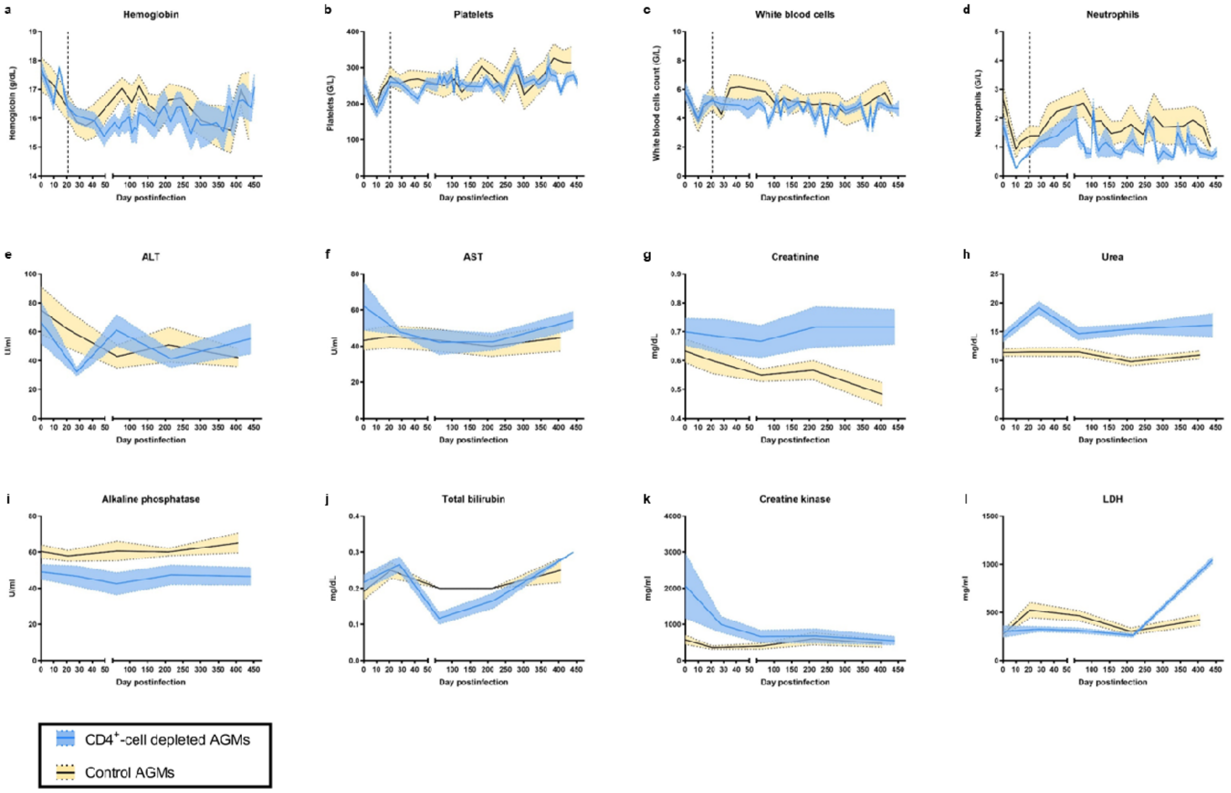

a-d. Evolution of different proteins and cell populations in peripheral blood of $CD4^{+}$-cell-depleted (blue) and control (yellow) AGMs during the follow-up: hemoglobin (a), platelets (b), leucocytes (c) and neutrophils (d). **e-l.** Evolution of different markers known to be associated with toxicity. Data shown in all panels are means (solid lines) ± standard error of the means (shaded regions within the dashed lines), with n=6 AGMs in both groups. Abbreviations: ALT: alanine aminotransferase; AST: aspartate aminotransferase; LDH: lactate dehydrogenase.

Supplementary Fig. S2. Dynamics of $CD4^+$ T-cell memory subsets

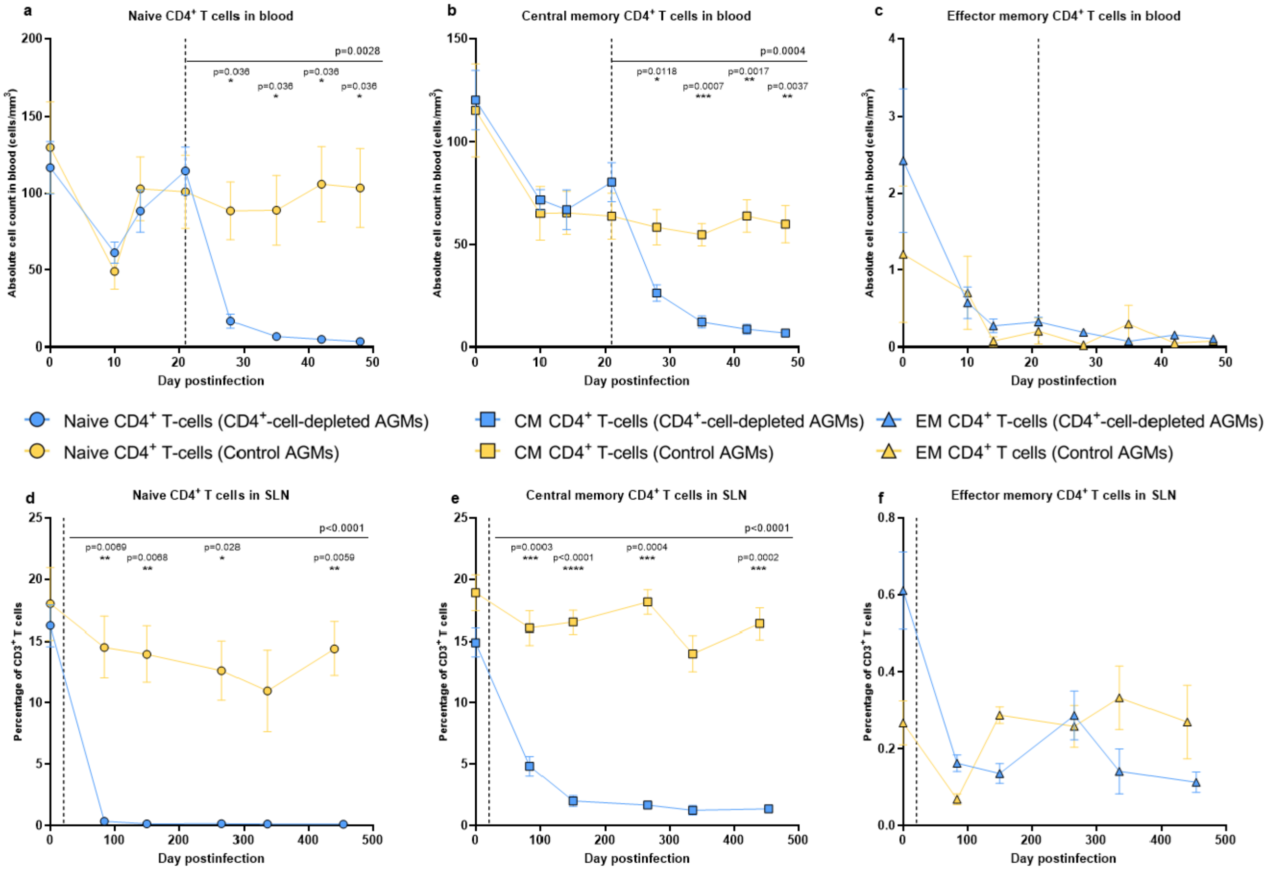

a-f. Dynamics of the different $CD4^+$ T-cell memory subsets were monitored in $CD4^+$-cell-depleted (blue) and control (yellow) AGMs, in peripheral blood (a-c) and sLNs (d-f). Naive ($CD28^+$, $CD95^{neg}$), central memory (CM; $CD28^+$, $CD95^+$) and effector memory (EM; $CD28^{neg}$, $CD95^+$) $CD4^+$ T-cells were identified by flow cytometry, and $CD4^+$ T-cell subsets were expressed as absolute cell counts per µL of peripheral blood (a-c) or percentages of $CD3^+$ T-cells in sLNs (d-f). The vertical dashed line indicates the initiation of the CD4-depleting antibody CD4R1 treatment. Values presented are means (solid lines) and standard error of the means (error bars), with n=6 AGMs in both groups. Differences between the two groups in the levels of memory subsets of $CD4^+$ T-cells in blood and sLNs after administering the $CD4^+$-cell-depleting antibody were assessed using mixed-effects models, with Holm-Šídák's correction for multiple comparisons. Mixed-effects model analyses revealed that $CD4^+$-cell depletion had a significant effect on the levels of naïve and central memory subsets of $CD4^+$ T-cells, and the corresponding p value is reported in the top right corner. Asterisks indicate timepoints for which differences were significant between the two groups, with *: $p<0.05$, **: $p<0.01$ and ***: $p<0.001$. Abbreviations: CM: central memory, EM: effector memory, sLN: superficial lymph node.

Supplementary Fig. S3. Polyfunctionality of the T-cell subsets in CD4⁺-cell-depleted AGMs

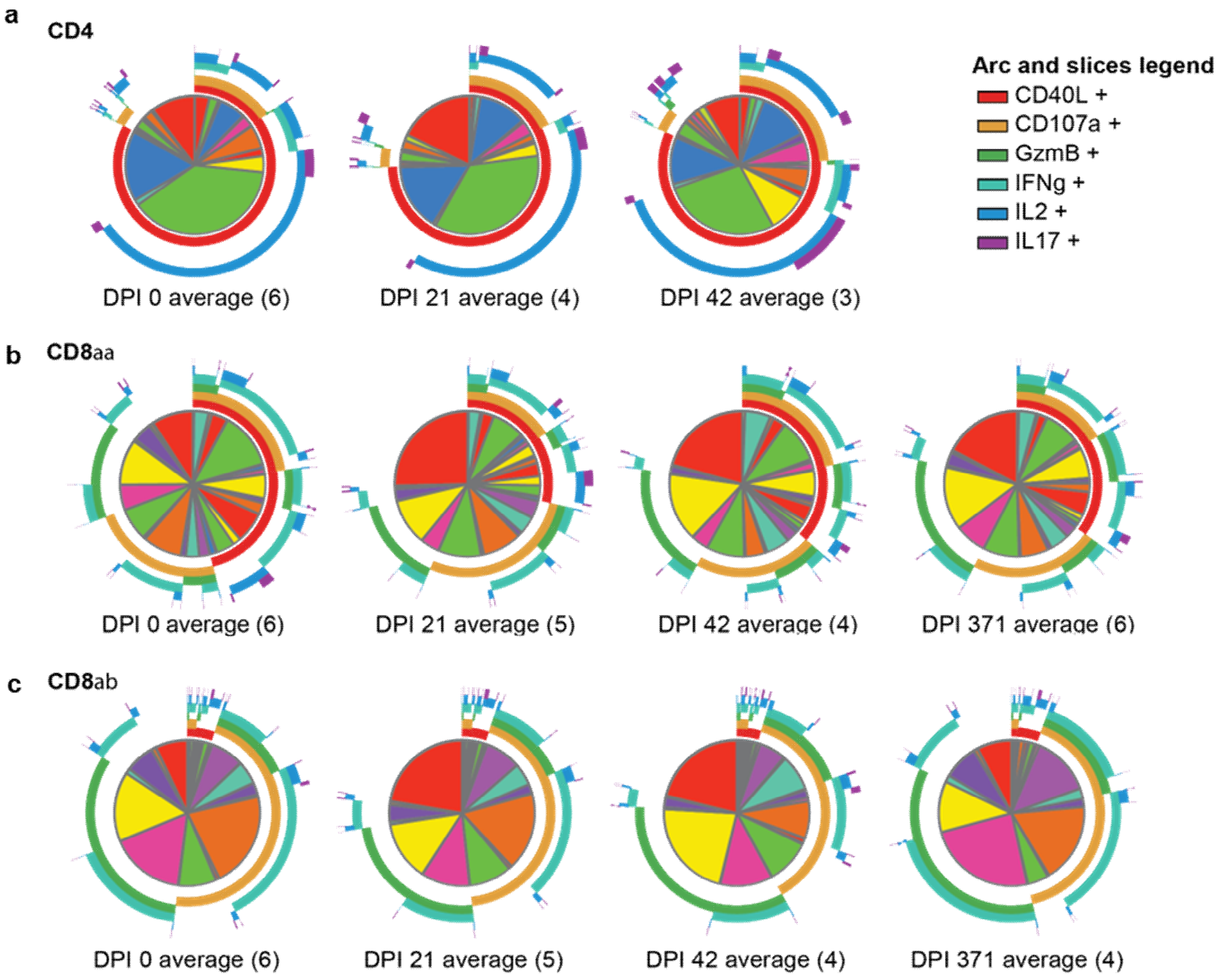

a-c. Polyfunctionality of T-cell subsets in $CD4^+$ T-cell-depleted AGMs was evaluated through the expression of six functional markers in PMA/ionomycin-stimulated T-cells using Boolean gating and averaged for the number of AGMs indicated in the parenthesis under each plot. Only populations with at least 200 cells were analyzed, with n=6 AGMs for each group. Due to low $CD4^+$ T-cell counts at 371 dpi, this T-cell subset could not be analyzed for this timepoint. The patterns of polyfunctionality differed significantly between $CD4^+$ (a) and either CD8αα (b) or CD8αβ (c) T-cell populations at each timepoint ($p<0.05$, partial permutation test).

Supplementary Fig. S4. Polyfunctionality of T-cell subsets in controls.

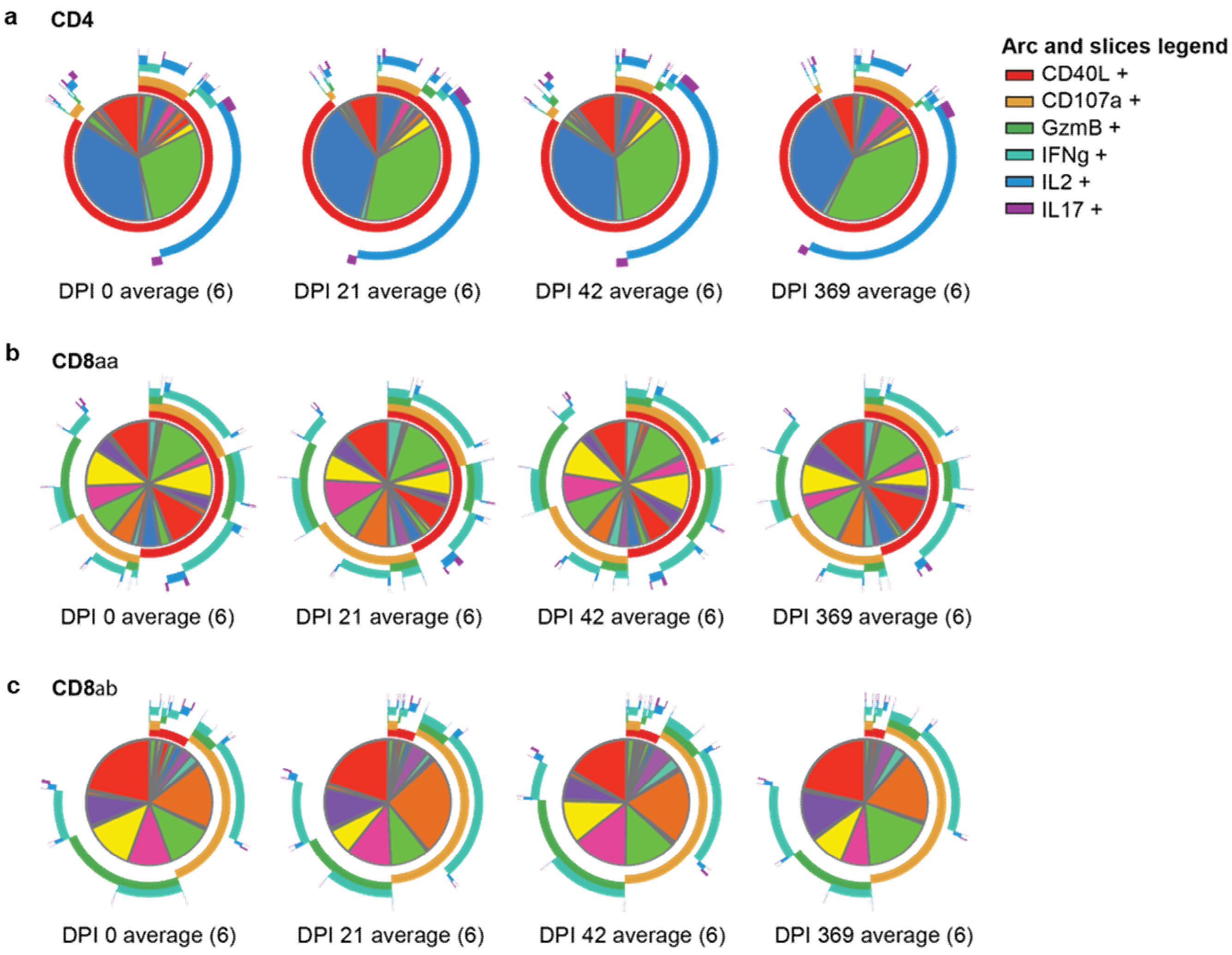

a-c. Polyfunctionality of T-cell subsets in controls was evaluated through the expression of six functional markers in PMA/ionomycin-stimulated T-cells using Boolean gating and averaged for the number of AGMs indicated in the parenthesis under each plot. Only populations with at least 200 cells were analyzed, with n=6 AGMs for each group. The patterns of polyfunctionality differed significantly between CD4^{+} (a) and either CD8αα (b) or CD8αβ (c) T-cell populations at each timepoint ($p<0.05$, partial permutation test).

Supplementary Fig. S5. Cell proliferation of CD8⁺ T-cells in the gastrointestinal tract and the liver

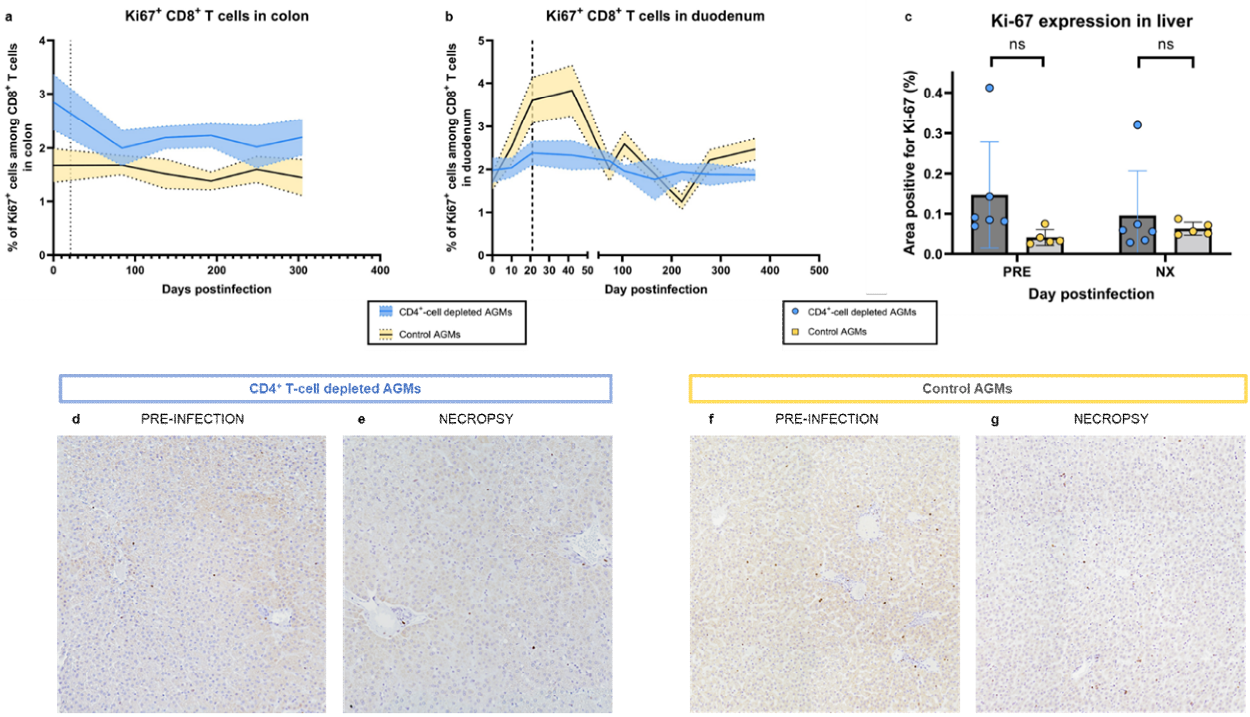

a-b. CD8⁺ T-cell proliferation in colon (a) and duodenal (b) biopsies was measured by flow cytometry, by assessing the expression of the intracellular marker Ki-67. Data presented in panels A and B are means (solid lines) and standard deviations of the means (shaded regions within the dashed lines) (n=6 AGMs for both groups). For panel c, data were analyzed with unpaired, two-sided, nonparametric Mann-Whitney tests, followed by Holm-Sidak's correction for multiple comparisons. **c-g.** Expression of Ki-67 in the liver of CD4⁺-cell-depleted AGMs (blue) and controls (yellow) was assessed by IHC. Data presented in panel c are means and standard deviations (n=6 animals for CD4⁺-cell-depleted AGMs and n=5 animals for controls). Representative IHC images of liver of CD4⁺-cell-depleted AGMs (d-e) and controls (f-g) stained for Ki-67 expression (brown) were taken at x400 magnification. Those images are representatives of images taken for the 6 AGMs of the group, with at least 20 images taken per animal.

Supplementary Fig. S6. Individual plasma viral loads of CD4^{+}-cell-depleted AGMs

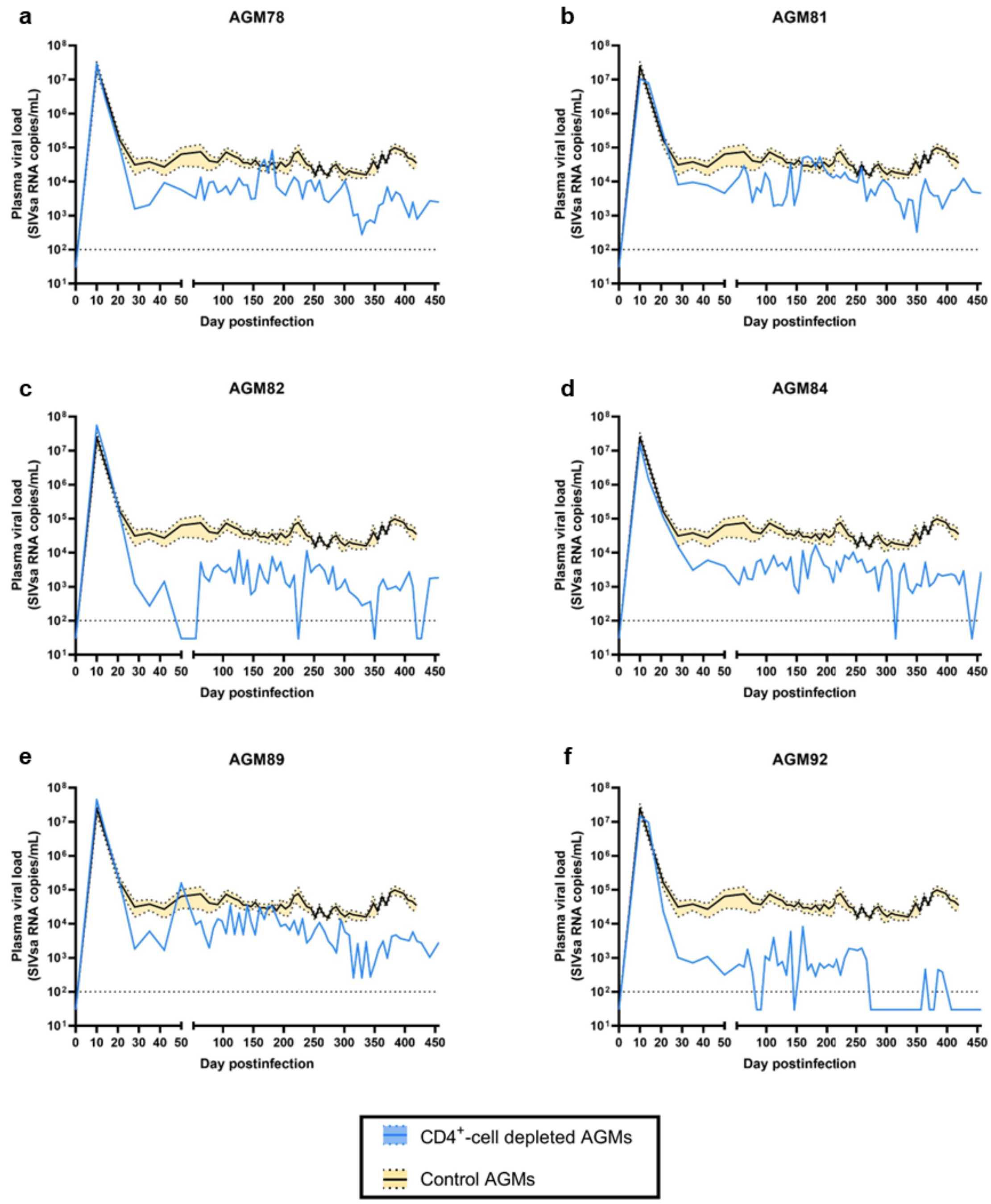

a-f. Plasma viral loads in each of the CD4^{+}-cell-depleted AGM (blue) throughout the follow-up. In all panels, mean (solid line) and standard error of the mean (shaded region within the dashed lines) of the plasma viral loads in the 6 control AGMs (yellow) are shown. The horizontal dashed line indicates the limit of quantification (100 copies/mL). For all samples, qRT-PCR were performed in duplicate.

Supplementary Fig. S7. Analyses of *env* sequences before and after CD4^{+}-cell depletion

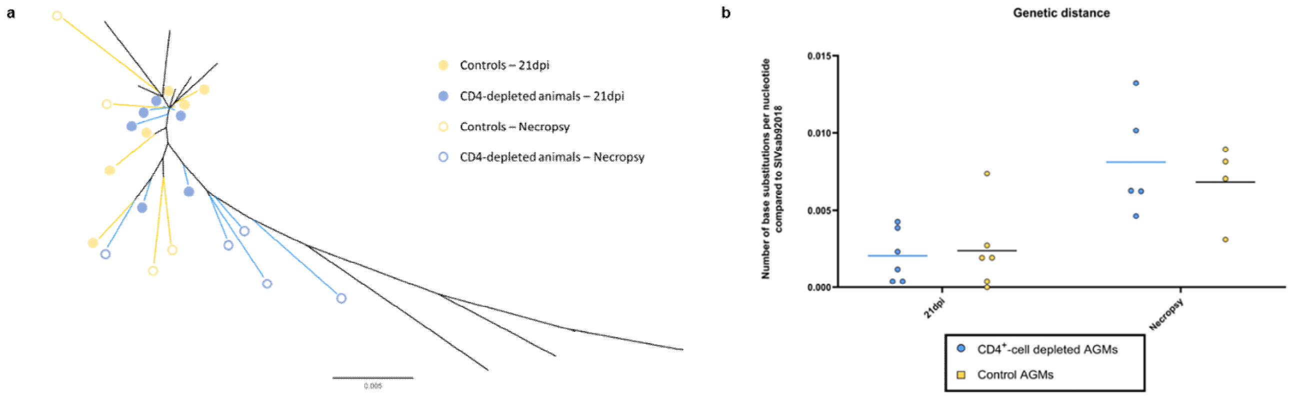

c

	V1 loop		V2 loop		V3 loop sequence																																		V5 loop	
Virus	141-143	156-158	171-173	190-192	1	2	3	4	5	6	7	8	9	10	11	12	13	14	15	16	17	18	19	20	21	22	23	24	25	26	27	28	29	30	31	32	33	34	V3 loop net charge	476-478	479-481
SIVsab92018.MH029382	N A T	N C S	N Y S	N R S	C	R	R	P	G	N	K	T	V	L	P	V	T	I	M	A	G	L	V	F	H	S	Q	K	Y	N	T	R	L	K	Q	A	W	C	6	N R T	N V T
Control AGM148 21dpi	...	...	...	...	.	.	.	.	.	.	.	.	.	.	.	.	.	.	.	.	.	.	.	.	.	.	.	.	.	.	.	.	.	.	.	.	.	.	6	...	...
Control AGM148 Necropsy	...	...	...	...	.	.	.	.	.	.	.	.	.	.	.	.	.	.	.	.	.	.	.	.	.	.	.	.	.	.	.	.	.	.	.	.	.	.	6	...	...
Control AGM151 21dpi	...	...	...	...	.	.	.	.	.	.	.	.	.	.	.	.	.	.	.	.	.	.	.	.	.	.	.	.	.	.	.	.	.	.	.	.	.	.	6	...	...
Control AGM154 21dpi	...	...	...	...	.	.	.	.	.	.	.	.	.	.	.	.	.	.	.	.	.	.	.	.	.	.	.	.	.	.	.	.	.	R	.	.	.	.	6	...	...
Control AGM155 21dpi	...	...	...	...	.	.	.	.	.	.	.	.	.	.	.	.	.	.	.	.	.	.	.	.	.	.	.	.	.	.	.	.	.	.	.	.	.	.	6	...	...
ControlAGM155 Necropsy	...	...	...	...	.	.	.	.	.	.	.	.	.	.	.	.	.	.	.	.	.	.	.	.	.	.	.	.	.	.	.	.	.	.	.	.	.	.	6	...	...
Control AGM156 21dpi	...	...	...	...	.	.	.	.	.	.	.	.	.	.	.	.	.	.	.	.	.	.	.	.	.	.	.	.	.	.	.	.	.	.	.	.	.	.	6	...	...
Control AGM156 Necropsy	...	...	...	.G.	.	.	.	.	.	.	.	.	.	.	.	.	.	.	.	.	.	.	.	.	.	.	.	.	.	.	.	.	.	.	.	.	.	.	6	...	...
Control AGM157 21dpi	...	...	...	...	.	.	.	.	.	.	.	.	.	.	.	.	.	.	.	.	.	.	.	.	.	.	.	.	.	.	.	.	.	.	.	.	.	.	6	...	...
Control AGM157 Necropsy	...	...	...	...	.	.	.	.	.	.	.	.	.	.	.	.	.	.	.	.	.	.	.	.	.	.	.	.	.	.	.	.	.	R	.	.	.	.	6	...	...
CD4-depleted AGM78 21dpi	...	...	...	...	.	.	.	.	.	.	.	.	.	.	.	.	.	.	.	.	.	.	.	.	.	.	.	.	.	.	.	.	.	.	.	.	.	.	6	...	...
CD4-depleted AGM78 Necropsy	...	...	...	.X.	.	.	.	.	.	.	.	.	.	.	.	.	.	.	.	.	.	.	.	.	.	.	.	.	.	.	.	.	.	.	.	.	.	.	6	...	...
CD4-depleted AGM81 21dpi	...	...	...	...	.	.	.	.	.	.	.	.	.	.	.	.	.	.	.	.	.	.	.	.	.	.	.	.	.	.	.	.	.	.	.	.	.	.	6	...	...
CD4-depleted AGM81 Necropsy	...	...	...	...	.	.	.	.	.	.	.	.	M	.	.	.	.	.	.	.	.	.	.	.	.	N	.	.	.	.	.	.	.	.	.	.	.	.	6	...	...
CD4-depleted AGM82 21dpi	...	...	...	...	.	.	.	.	.	.	.	.	.	.	.	.	.	.	.	.	.	.	.	.	.	.	.	.	.	.	.	.	.	.	.	.	.	.	6	...	...
CD4-depleted AGM82 Necropsy	...	...	...	...	.	.	.	.	.	.	.	.	.	.	.	.	.	.	.	.	.	.	.	.	.	.	.	.	.	.	.	.	.	.	.	.	.	.	6	...	...
CD4-depleted AGM84 21dpi	...	...	...	...	.	.	.	.	.	.	.	.	.	.	.	.	.	.	.	.	.	.	.	.	.	.	.	.	.	.	.	.	.	.	.	.	.	.	6	...	...
CD4-depleted AGM84 Necropsy	...	...	...	.G.	.	.	.	.	.	.	.	.	.	.	.	.	.	.	.	.	.	.	.	.	.	.	.	.	.	.	.	.	.	.	.	.	.	.	6	...	...
CD4-depleted AGM89 21dpi	...	...	...	...	.	.	.	.	.	.	.	.	.	.	.	.	.	.	.	.	.	.	.	.	.	.	.	.	.	.	.	.	.	.	.	.	.	.	6	...	...
CD4-depleted AGM89 Necropsy	...	...	...	...	.	.	.	.	.	.	.	.	.	.	.	.	.	.	.	.	.	.	.	.	.	.	.	.	.	.	.	.	.	.	.	.	.	.	6	...	...
CD4-depleted AGM92 14dpi	...	...	...	...	.	.	.	.	.	.	.	.	.	.	.	.	.	.	.	.	.	.	.	.	.	.	.	.	.	.	.	.	.	.	.	.	.	.	6	...	...
SAB.SN.2005.Sab92018ivTF.HQ378594	...	...	...	...	.	.	.	.	.	.	.	.	.	.	.	.	.	.	.	.	.	.	.	.	.	.	.	.	.	.	.	.	.	.	.	.	.	.	6	...	...
SAB.DE.2012.14623.2012.TP10.MF775060	...	...	...	D--	.	.	.	.	.	.	.	.	.	.	.	.	.	.	.	.	.	.	.	.	.	.	.	.	.	.	.	.	.	.	.	.	.	.	6	...	...
SAB.US.2009.BA38_A11.KP896179	...	...	...	.G.	.	.	.	.	.	.	.	.	.	.	.	.	.	.	.	.	.	.	.	.	.	.	.	.	.	.	.	.	.	.	.	.	.	.	6	...	...
SAB.US.2009.P373_1.KP896191	...	...	...	...	.	.	.	.	.	.	.	.	.	.	.	.	.	.	.	.	.	.	.	.	.	.	.	.	.	.	.	.	.	.	.	.	.	.	6	...	...
SAB.US.2009.RM88_1.KP896233	...	...	...	...	.	.	.	.	.	.	.	.	.	.	.	.	.	.	.	.	.	.	.	.	.	.	.	.	.	.	.	.	.	.	.	.	.	.	6	...	...
SAB.US.2009.RM89_1.KP896272	...	...	...	...	.	.	.	.	.	.	.	.	.	.	.	.	.	.	.	.	.	.	.	.	.	.	.	.	.	.	.	.	.	.	.	.	.	.	6	...	...
SAB.US.2009.V492_1.KP896161	...	...	...	.G.	.	.	.	.	.	.	.	.	.	.	.	.	.	.	.	.	.	.	.	.	.	.	.	.	.	.	.	.	.	.	.	.	.	.	6	...	...
SAB.US.2011.Plasma_AGM91_5m_A.MH029383	...	...	...	---	.	.	.	.	.	.	.	.	.	.	.	.	.	.	.	.	.	.	.	.	.	.	.	.	.	.	.	.	.	.	.	.	.	.	6	...	...
SAB.US.2011.Plasma_AGM92_5m_AB.MH029401	...	...	...	...	.	.	.	.	.	.	.	.	.	.	.	.	.	.	.	.	.	.	.	.	.	.	.	.	.	.	.	.	.	.	.	.	.	.	6	...	...
SAB.US.2011.Plasma_AGM93_5m_A.MH029418	...	...	...	.G.	.	.	.	.	.	.	.	.	.	.	.	.	.	.	.	.	.	.	.	.	.	.	.	.	.	.	.	.	.	.	.	.	.	.	6	...	...
SAB.US.2011.Plasma_AGM94_5m_AB.MH029435	...	...	...	T..	.	.	.	.	.	.	.	.	.	.	.	.	.	.	.	.	.	.	.	.	.	.	.	.	.	.	.	.	.	.	.	.	.	.	6	...	...
SAB.US.2012.Plasma_AGM91_1y_A.MH029454	...	...	...	.G.	.	.	.	.	.	.	.	.	.	.	.	.	.	.	.	.	.	.	.	.	.	.	.	.	.	.	.	.	.	.	.	.	.	.	6	...	...
SAB.US.2012.Plasma_AGM92_1y_A.MH029468	...	...	...	.--	.	.	.	.	.	.	.	.	.	.	.	.	.	.	.	.	.	.	.	.	.	.	.	.	.	.	.	.	.	R	.	.	.	.	6	...	...
SAB.US.2012.Plasma_AGM94_1y_B.MH029492	...	...	...	--.	.	.	.	.	.	.	.	.	.	.	.	.	.	.	.	.	.	.	.	.	.	.	.	.	.	.	.	.	.	.	.	.	.	.	6	...	...

a. A phylogenetic tree was built using a dataset of 36 SIVsab92018 *env* sequences (21 from the AGMs included in this study, 12 obtained at 21 dpi and 9 obtained at necropsy, plus 15 sequences retrieved from the Los Alamos National Laboratory Database (http://www.hiv.lanl.gov/). The phylogenetic tree was built with PhyML 3.0, using a maximum likelihood method with a General Time Reversible (GTR) model, with 500 bootstraps. The phylogenetic tree with the highest log likelihood is displayed. Branch length represents the genetic distance. **b.** Evolutionary divergence between each *env* sequence and the parental strain, SIVsab92018. Genetic distance (i.e., the number of base substitutions per site between each *env* sequence and the parental strain, SIVsab92018) was calculated using the Maximum Composite Likelihood model in MEGA11. **c.** Alignment of *env* amino acid sequences of the 36 SIVsab strains included in the phylogenetic analysis. Potential asparagine-linked glycosylation sites (NGS) in the V1, V2 and V5 loops, the entire V3 loop sequence and the net charge of this V3 loop are

reported in this table, as they are determinants of either a switch to CD4-independence (for NGS) or to a X4 tropism (for the V3 loop sequence and its net charge). Potential NGS are defined as N-X-S/T motifs, with N, S and T being asparagine, serine and threonine, respectively, and X is any amino acid except proline. Dots represent positions for which the amino acid do not differ from the one in the reference sequence (SIVsab92018), while dashes represent position for which there is a deletion of the amino acid present in the reference strain. Positions for which a loss of the NGS is predicted or a nonsynonymous mutation in the V3 loop is observed are indicated in bold.

Supplementary Fig. S8. Gating strategy

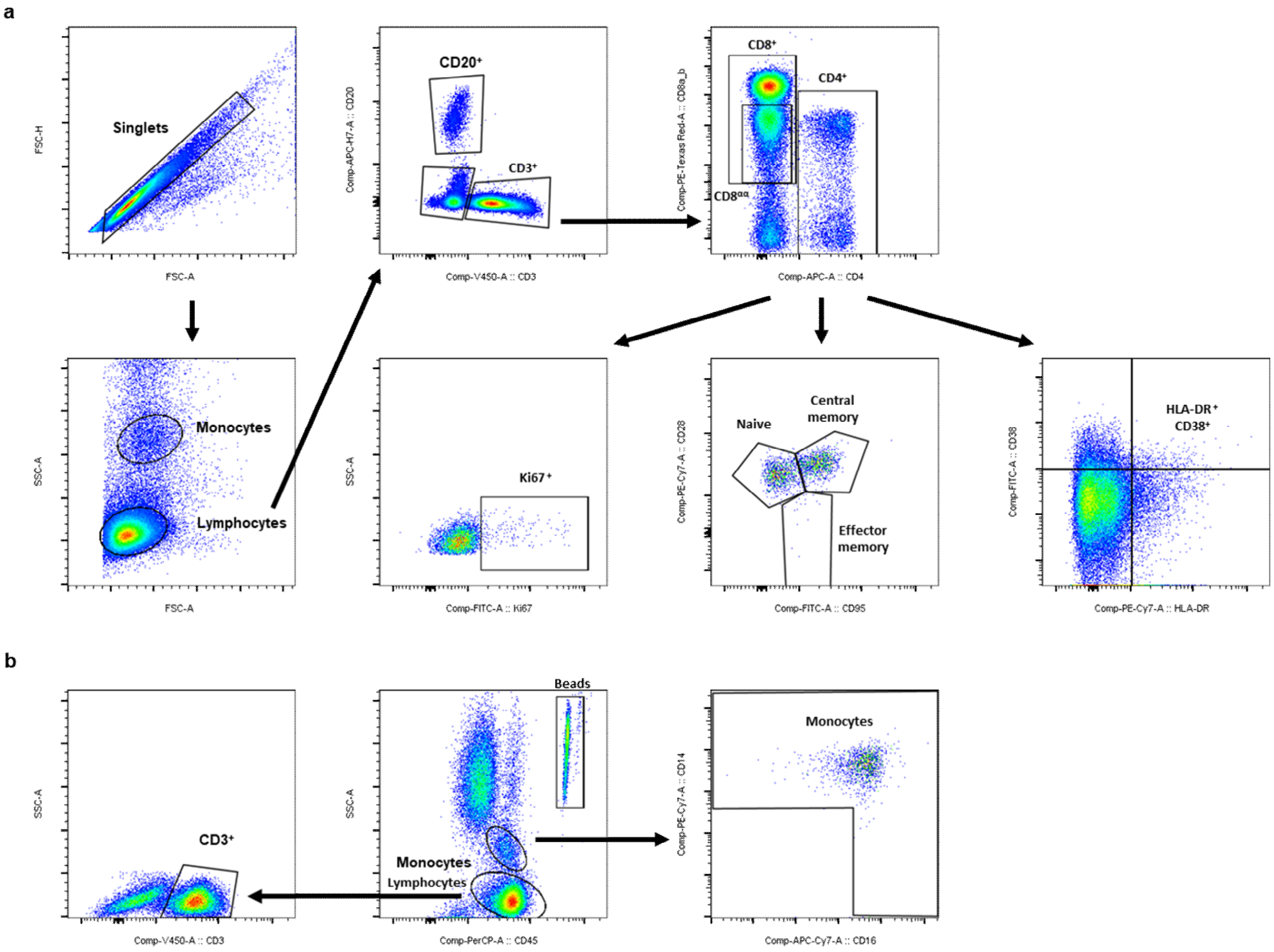

a. Gating strategy used to delineate primary T-cell populations: CD3$^+$, CD4$^+$, CD8$^+$, CD8$^{\alpha\alpha}$ T-cells, as well as B-cells (CD20$^+$). Gating strategies to delineate the secondary T-cell populations (effector memory, central memory and naïve), Ki-67$^+$ T cells, and HLA-DR$^+$ CD38$^+$ T-cells are also shown. Each of those secondary T-cell populations were determined with a different staining. All plots shown for secondary T-cell populations, Ki-67$^+$ T cells, and HLA-DR$^+$ CD38$^+$ T-cells were gated on CD4$^+$ T-cells, but the same gating strategies were used for CD8$^+$ T-cells. Similar gating strategies were applied for blood, lymph nodes and intestinal biopsies. **b.** Gating strategy for the Trucount tubes to quantify the number of circulating CD3$^+$ T-cells and monocytes.

Supplementary tables.

Supplementary Table 1. Results of partial permutation tests performed in SPICE for $CD4^{+}$-cell-depleted AGMs.

$p<0.05$ indicates profiles are significantly different.

Permutation Test
DPI 0

#	1	2	3
1		0.0007	0.0001
2	0.0007		0.0016
3	0.0001	0.0016	

1 = CD4, 2 = CD8αα, 3 = CD8αβ

Permutation Test
DPI 21

#	1	2	3
1		0.0063	0.0068
2	0.0063		0.4553
3	0.0068	0.4553	

1 = CD4, 2 = CD8αα, 3 = CD8αβ

Permutation Test
DPI 42

#	1	2	3
1		0.0338	0.0217
2	0.0338		0.5055
3	0.0217	0.5055	

1 = CD4, 2 = CD8αα, 3 = CD8αβ

Permutation Test
DPI 371

#	1	2
1		0.0218
2	0.0218	

1 = CD8αα, 2 = CD8αβ

Permutation Test
CD4

#	1	2	3
1		0.3630	0.1113
2	0.3630		0.5053
3	0.1113	0.5053	

1 = DPI 0, 2 = DPI 21, 3 = DP1 42

Permutation Test
CD8aa

#	1	2	3	4
1		0.1214	0.3735	0.4844
2	0.1214		0.8094	0.5509
3	0.3735	0.8094		0.9517
4	0.4844	0.5509	0.9517	

1 = DPI 0, 2 = DPI 21, 3 = DP1 42, 4 = DPI 371

Permutation Test
CD8ab

#	1	2	3	4
1		0.3349	0.1644	0.5425
2	0.3349		0.7160	0.2488
3	0.1644	0.7160		0.1796
4	0.5425	0.2488	0.1796	

1 = DPI 0, 2 = DPI 21, 3 = DP1 42, 4 = DPI 371

Supplementary Table 2. Results of partial permutation tests performed in SPICE for control animals.

Test for significant differences between timepoints within each T-cell subset:

1=D0, 2=D21, 3=D42, 4=D369

Permutation Test
CD4

#	1	2	3	4
1		0.5585	0.6955	0.2833
2	0.5585		0.9327	0.9122
3	0.6955	0.9327		0.6328
4	0.2833	0.9122	0.6328	

Permutation Test
CD8aa

#	1	2	3	4
1		0.7167	0.7218	0.6878
2	0.7167		0.7689	0.6392
3	0.7218	0.7689		0.4104
4	0.6878	0.6392	0.4104	

Permutation Test
CD8ab

#	1	2	3	4
1		0.6746	0.8638	0.7958
2	0.6746		0.6686	0.5824
3	0.8638	0.6686		0.4812
4	0.7958	0.5824	0.4812	

Test for significant differences between T-cell subsets at each timepoint

1=CD4, 2 = CD8αα, 3 = CD8αβ

Permutation Test
D0

#	1	2	3
1		0.0019	0.0026
2	0.0019		0.0011
3	0.0026	0.0011	

Permutation Test
D21

#	1	2	3
1		0.0010	0.0020
2	0.0010		0.0028
3	0.0020	0.0028	

Permutation Test
D42

#	1	2	3
1		0.0013	0.0010
2	0.0013		0.0005
3	0.0010	0.0005	

Permutation Test
D369

#	1	2	3
1		0.0026	0.0016
2	0.0026		0.0027
3	0.0016	0.0027	
