# Supplementary material for: Prolonged experimental CD4+ T-cell depletion does not cause disease progression in SIV-infected African green monkeys
Source: Nat Commun. 2023 Feb 22;14:979. doi: 10.1038/s41467-023-36379-2 (PMC9946951; doi:10.1038/s41467-023-36379-2)
Supplement: Supplementary file 3 — Reporting Summary [file 41467_2023_36379_MOESM3_ESM.pdf]

nature portfolio

Corresponding author(s): Pandrea Ivona

Last updated by author(s): Dec 4, 2022

Reporting Summary

Nature Portfolio wishes to improve the reproducibility of the work that we publish. This form provides structure for consistency and transparency in reporting. For further information on Nature Portfolio policies, see our Editorial Policies and the Editorial Policy Checklist.

Statistics

For all statistical analyses, confirm that the following items are present in the figure legend, table legend, main text, or Methods section.

n/a	Confirmed	
☐	☒	The exact sample size (*n*) for each experimental group/condition, given as a discrete number and unit of measurement
☐	☒	A statement on whether measurements were taken from distinct samples or whether the same sample was measured repeatedly
☐	☒	The statistical test(s) used AND whether they are one- or two-sided<br>*Only common tests should be described solely by name; describe more complex techniques in the Methods section.*
☒	☐	A description of all covariates tested
☐	☒	A description of any assumptions or corrections, such as tests of normality and adjustment for multiple comparisons
☐	☒	A full description of the statistical parameters including central tendency (e.g. means) or other basic estimates (e.g. regression coefficient) AND variation (e.g. standard deviation) or associated estimates of uncertainty (e.g. confidence intervals)
☐	☒	For null hypothesis testing, the test statistic (e.g. *F*, *t*, *r*) with confidence intervals, effect sizes, degrees of freedom and *P* value noted<br>*Give P values as exact values whenever suitable.*
☒	☐	For Bayesian analysis, information on the choice of priors and Markov chain Monte Carlo settings
☒	☐	For hierarchical and complex designs, identification of the appropriate level for tests and full reporting of outcomes
☒	☐	Estimates of effect sizes (e.g. Cohen's *d*, Pearson's *r*), indicating how they were calculated

Our web collection on statistics for biologists contains articles on many of the points above.

Software and code

Policy information about availability of computer code

Data collection	Flow cytometry acquisitions were performed on an LSR II or a LSR Fortessa flow cytometer (BD Biosciences). Plasma viral loads and levels of cell-associated viral RNA and DNA were measured with RT-PCR or PCR performed on a 7900HT Fast Real Time System (Applied Biosystems).
Data analysis	Microsoft Excel version 2208<br>GraphPad Prism 9.3.1<br>FlowJo v10.7.1 and v10.8.0<br>SPICE6<br>PhyML 3.0<br>MEGA11

For manuscripts utilizing custom algorithms or software that are central to the research but not yet described in published literature, software must be made available to editors and reviewers. We strongly encourage code deposition in a community repository (e.g. GitHub). See the Nature Portfolio guidelines for submitting code & software for further information.

March 2021

Data

Policy information about availability of data

All manuscripts must include a data availability statement. This statement should provide the following information, where applicable:

- Accession codes, unique identifiers, or web links for publicly available datasets
- A description of any restrictions on data availability
- For clinical datasets or third party data, please ensure that the statement adheres to our policy

Data availability: Source data are provided with this paper (https://doi.org/10.6084/m9.figshare.21200539.v1). A dataset composed of 36 SIVsab env sequences (21 obtained in this study, plus 15 retrieved from the Los Alamos National Laboratory website) was used for phylogenetic analysis. The 21 env sequences obtained in this study are accessible on Genbank (accession numbers: OP491432-OP491452).

Human research participants

Policy information about studies involving human research participants and Sex and Gender in Research.

Reporting on sex and gender	N/A
Population characteristics	N/A
Recruitment	N/A
Ethics oversight	N/A

Note that full information on the approval of the study protocol must also be provided in the manuscript.

Field-specific reporting

Please select the one below that is the best fit for your research. If you are not sure, read the appropriate sections before making your selection.

[x] Life sciences [ ] Behavioural & social sciences [ ] Ecological, evolutionary & environmental sciences

For a reference copy of the document with all sections, see nature.com/documents/nr-reporting-summary-flat.pdf

Life sciences study design

All studies must disclose on these points even when the disclosure is negative.

Sample size	Twelve male African Green Monkeys (AGMs) were included in this study. All AGMs were housed at the University of Pittsburgh (PA, USA), according to the Association and Accreditation of Laboratory Animal Care (AAALAC) guidelines. Animal experiments were approved by the University of Pittsburgh Institutional Animal Care and Use Committee (IACUC) (protocol #19074902).<br>No formal sample size calculation was performed. A sample size of N=12 (N=6 animals per study group and N=6 animals per control group) was selected based on our experience to date. In our previous studies (He et al., JCI, 2018; Schechter et al., Sci Transl. Med, 2017; Pandrea, JVI 2008; Pandrea, JVI 2006), we were able to detect significant differences in the main parameters investigated here with similar or even lower experimental group sizes.
Data exclusions	No data was excluded during this study but data are missing for some timepoints, either due to a lack of tissues (for IHC and PCR assays) or due to an insufficient number of cells (for flow cytometry experiments).
Replication	Due to high costs and the fact that all assays were done on all 12 animals, we have not performed replication analysis. However, we have used reagents that have been widely used in the field. Moreover, we had values at baseline (for CD4 T cells, viral loads and levels of inflammation) that were in the range of what was previously reported for SIVsab-infected AGMs in the literature.<br>Longitudinal samples were used for this study. We compared study animals with themselves and with control animals, over the entire followup. When evaluating inflammation, gut integrity, immune activation, at least 2 different techniques were used. For biomarkers, when possible, samples from the same animal were tested in the same round to limit inter-batch variation. For PCR assays (viral load, cell-associated viral RNA and DNA), samples were tested in duplicate.
Randomization	Those animals were not randomly allocated, but before initiating CD4-depleting antibody, plasma viral loads were quantified to verify that all animals were within the same range.
Blinding	No, investigators were not blinded during data acquisition or analysis. As all lab members are involved in cell separation and preparation of the dilutions of the CD4-depleting antibody, it was not feasible to keep everyone blinded as the lymphocytes numbers were drastically different between the 2 groups. The person running the PCR assays was not involved in cell separation, and was thus kept blinded.

Reporting for specific materials, systems and methods

We require information from authors about some types of materials, experimental systems and methods used in many studies. Here, indicate whether each material, system or method listed is relevant to your study. If you are not sure if a list item applies to your research, read the appropriate section before selecting a response.

Materials & experimental systems

n/a	Involved in the study	
☐	☒	Antibodies
☒	☐	Eukaryotic cell lines
☒	☐	Palaeontology and archaeology
☐	☒	Animals and other organisms
☒	☐	Clinical data
☒	☐	Dual use research of concern

Methods

n/a	Involved in the study	
☒	☐	ChIP-seq
☐	☒	Flow cytometry
☒	☐	MRI-based neuroimaging

Antibodies

Antibodies used

The following antibodies were used to determine the absolute cell counts in peripheral blood, with Trucount tubes: CD45 (PerCP-Cy5-5, 3µL, clone D058-1283), CD3 (V450, 2µL,SP34-2), CD14 (PE-Cy7, 4µL, M5E2), CD16 (APC-Cy7, 2µL, 3G8) and CD163 (APC, 5µL,GHI/61).
For other flow cytometry experiments, the following antibodies were used: Annexin V (5µL,FITC), CCR5 (10µL, PE), CD3 (V450, 2µL, clone SP34-2), CD4 (APC, 2.5µL, L200), CD8 (PE-CF594, 3µL, RPA-T8), CD14 (PE-Cy7, 4µL, M5E2), CD16 (APC-Cy7, 4µL, 3G8), CD163 (APC, 5µL, GHI/61), CD20 (APC-H7, 4µL, 2H7), CD28 (PE-Cy7, 3µL, CD28.2), CD38 (FITC, 15µL, AT-1) (Stemcell), CD69 (APC-H7, 5µL, FN50), CD95 (FITC, 15µL, DX2), HLA-DR (PE-Cy7, 3µL, L243), Ki-67 (FITC or PE, 20µL, B56), Live/Dead Aqua (200µL of a 1:500 dilution, Thermofisher).
For intracellular cytokine staining, the following antibodies were used: CD107a (BV711, 5µL, clone H4A3), Live/Dead Aqua (1µL), CD3 (ALX700, 3µL, SP34-2), CD4 (BV605, 5µL, L200), CD8 (PerCP-Cy5.5, 5µL, RPA-T8), CD28 (ECD, 5µL, CD28.2), CD95 (Cy5PE, 1µL, DX2), FoxP3 (Alx488, 5µL, 206D), GranzymeB (PE, 0.25µL, GB11), IFN (Cy7PE, 0.5µL, 4S.B3), IL-2 (BV785, 5µL, MQ1-17H12), IL-17 (e450, 5µL, eBio64DEC17), and CD40L (APC-e780, 5µL, 24-31).
For immunohistochemistry experiments, the following primary antibodies were used (200µL of a 1:100 dilution for all): Ki-67 (mouse monoclonal antibody, clone MIB-1, Dako), claudin-3 (rabbit polyclonal antibody, RB-9251-P1, Thermo Fisher), LPS (mouse monoclonal antibody, clone WN1 222-5, Hycult Biotech, USA).

Validation

All antibodies used were either validated to be human reactive with cross-reactivity for African Green Monkeys (NHP Reagent Resource, https://www.nhpreagents.org/ReactivityDatabase) or they were validated in previous studies :
Raehtz KD et al. African green monkeys avoid SIV disease progression by preventing intestinal dysfunction and maintaining mucosal barrier integrity. PLoS Pathog. 2020 Mar 2;16(3):e1008333.
He T et al. High-fat diet exacerbates SIV pathogenesis and accelerates disease progression. J Clin Invest. 2019 Dec 2;129 (12):5474-5488.

Animals and other research organisms

Policy information about studies involving animals; ARRIVE guidelines recommended for reporting animal research, and Sex and Gender in Research

Laboratory animals

Twelve African green monkeys were included in this study. Those AGMs were all adult males. The animals' age was determined by the veterinarians based on phenotypic criteria.

Wild animals

N/A

Reporting on sex

Sex was not considered in our study design, as sex is not known to have a major impact on CD4+ T-cell levels (Pandrea I et al., Impact of viral factors on very early in vivo replication profiles in simian immunodeficiency virus SIVagm-infected African green monkeys. J Virol. 2005 May;79(10):6249-59) and plasma viral loads (Ma D et al., Factors associated with siman immunodeficiency virus transmission in a natural African nonhuman primate host in the wild. J Virol. 2014 May;88(10):5687-705.) in SIVsab-infected AGMs.

Field-collected samples

NA

Ethics oversight

University of Pittsburgh Institutional Animal Care and Use Committee (IACUC) (protocol #19074902)

Note that full information on the approval of the study protocol must also be provided in the manuscript.

Flow Cytometry

Plots

Confirm that:

☒ The axis labels state the marker and fluorochrome used (e.g. CD4-FITC).

☒ The axis scales are clearly visible. Include numbers along axes only for bottom left plot of group (a 'group' is an analysis of identical markers).

☒ All plots are contour plots with outliers or pseudocolor plots.

☒ A numerical value for number of cells or percentage (with statistics) is provided.

Methodology

Sample preparation

All samples were taken from SIVsab-infected AGMs. Immunophenotyping was performed on freshly isolated cells, except for intracellular cytokine staining that was performed on cryopreserved PBMC.

Immunophenotyping :
White blood cells from blood and mononuclear cells isolated from intestinal biopsies and LNs were immunophenotyped by flow cytometry, as described.67 First, TruCount staining was performed on 50 µL of whole blood, using CD45 (PerCP-Cy5-5, 3µL, clone D058-1283), CD3 (V450, 2µL,SP34-2), CD14 (PE-Cy7, 4µL, M5E2), CD16 (APC-Cy7, 2µL, 3G8) and CD163 (APC, 5µL,GHI/61) antibodies. This allowed us to precisely quantify circulating CD45+ cells, T cells and monocytes. The exact counts of CD4+ and CD8+ T-cells were determined by multiplying the exact number of CD3+ T-cells by the percentage of CD4+ or CD8+ cells among CD3+ T-cells that were determined in a different staining. CD14 and CD16 were used to identify circulating monocyte subsets. Whole peripheral blood (100µL) and cells isolated from tissues were stained with fluorescently-labeled antibodies (all purchased from BD Bioscience, San Jose, CA, USA, unless noted otherwise): Annexin V (5µL,FITC), CCR5 (10µL, PE), CD3 (V450, 2µL, clone SP34-2), CD4 (APC, 2.5µL, L200), CD8 (PE-CF594, 3µL, RPA-T8), CD14 (PE-Cy7, 4µL, M5E2), CD16 (APC-Cy7, 4µL, 3G8), CD163 (APC, 5µL, GHI/61), CD20 (APC-H7, 4µL, 2H7), CD28 (PE-Cy7, 3µL, CD28.2), CD38 (FITC, 15µL, AT-1) (Stemcell), CD69 (APC-H7, 5µL, FN50), CD95 (FITC, 15µL, DX2), HLA-DR (PE-Cy7, 3µL, L243), Ki-67 (FITC or PE, 20µL, B56), Live/Dead Aqua (200µL of a 1:500 dilution, Thermofisher). For Ki-67 staining, cells were fixed, permeabilized with 1X BD Fix/Perm, then stained for Ki-67. All cells were washed then fixed with BD Fix before being analyzed.

Intracellular cytokine staining:
Cryopreserved cells were thawed, washed, and split into two tubes, each containing approximately 2x106 cells in 1 mL of complete RPMI media. Cells in one tube were stimulated with 5 ng/ml PMA and 1 µg/ml ionomycin. Both tubes were treated with 10 ng/ml of brefeldin A, 1 µl/ml monensin (BD GolgiStop), and CD107a (BV711, 5µL, clone H4A3) for 16-18 hours at 37° C. Cells were washed twice in PBS and stained with fixable Live/Dead Aqua (1µL), CD3 (ALX700, 3µL, SP34-2), CD4 (BV605, 5µL, L200), CD8 (PerCP-Cy5.5, 5µL, RPA-T8), CD28 (ECD, 5µL, CD28.2) and CD95 (Cy5PE, 1µL, DX2) for 20 mins at 4°C. Cells were washed with PBS and permeabilized with Foxp3 Perm Solution (eBioscience) for 1 hour at 4°C. Cells were washed twice with 2 mL FoxP3 Perm Wash Buffer Solution (eBioscience) and stained with FoxP3 (Alx488, 5µL, 206D), GranzymeB (PE, 0.25µL, GB11), IFN (Cy7PE, 0.5µL, 4S.B3), IL-2 (BV785, 5µL, MQ1-17H12), IL-17 (e450, 5µL, eBio64DEC17), and CD40L (APC-e780, 5µL, 24-31) for 30 mins at 4°C. Cells were washed twice with 2 mL FoxP3 Perm Wash Buffer Solution and fixed in 1% PFA before being analyzed on a LSR Fortessa.

Instrument

LSR II and LSR Fortessa flow cytometers

Software

Analyses of flow cytometry data were performed on FlowJo® v10.7.1 and FlowJo® v10.8.0 (Treestar).

Cell population abundance

No cell sorting was performed during this study. For peripheral blood and lymph nodes, at least 100,000 lymphocytes were recorded, and at least 10,000 lymphocytes for intestinal biopsies.

Gating strategy

The gating strategy is detailed in the supplementary figure 8. Mononuclear cells were defined as being on the diagonal of FSC-H versus FSC-A. Monocytes and lymphocytes were identified by using SSC-A versus FSC-A. Expression of CD20 and CD3 were used to discriminate between B and T cells. CD4+ and CD8+ T cells were gated on CD3+ T cells.

☒ Tick this box to confirm that a figure exemplifying the gating strategy is provided in the Supplementary Information.
